# CNN Multi-Position Wearable Sensor Human Activity Recognition Used in Basketball Training

**DOI:** 10.1155/2022/9918143

**Published:** 2022-09-19

**Authors:** Biao Tang, Wei Guan

**Affiliations:** ^1^Ministry of Sports and Arts, Hunan Modern Logistics Vocational and Technical College, Changsha 410007, Hunan, China; ^2^Hunan International Business Vocational College, Changsha 410201, Hunan, China

## Abstract

With the development trend of artificial intelligence technology and the popularization of wearable sensors, human activity recognition based on sensor data information has received widespread attention and has great application value. In order to better optimize the network structure and reduce the total number of main training parameters in the convolutional layer, a convolutional network entity model based on shared resources of main parameters is clearly proposed. We analyzed the CNN multi-position wearable sensor human activity recognition used in basketball training. According to the entity model of the main parameters of shared resources, the effectiveness of the entity model is verified from both the total number of sensors and the accuracy of single-class recognition. In addition to maintaining the actual effect of recognition, the main training parameters are also reduced. The simulation results verify the actual effect of the SVM algorithm and motion simulation of the convolutional network entity model. On this basis, scientific research physical exercise methods are selected to reasonably ensure the smooth progress of appropriate physical exercise at a certain level, improve the quality of training and the actual effect.

## 1. Introduction

Basketball is a new event in crowd sports. In short, it is a confrontation between shooting and scoring. For confrontation, the foundation must be established on the basic level of human energy and fitness exercise rate, as well as the reaction ability of the brain and limbs. However, under normal circumstances, human energy and fitness exercise rate occupy key positions and functions in basketball games. To some extent, it can immediately endanger the level of basketball test scores. Only by making full use of the advantages of fitness exercise speed can it have a beneficial impact on the competition situation [1]. Whether you are in a preemptive position or breaking through the line of defense, you can create an advantage. Through the analysis of the characteristics of the new basketball project, it can be seen that manpower and sports are the key to resisting the game. Therefore, as a basketball coach, in the whole process of training athletes, we must not only pay attention to the training of technical talents, but also improve the training of athletes' physical fitness and fitness speed. Only in this way can players get higher test scores in actual combat, which is why the key to improving physical training methods in the new basketball training lies in [2]. With the continuous advancement and development of science and technology in the current era, the application of sensors has become more and more extensive, and has been gradually applied to various fields, especially the training of athletes [3]. In the training of athletes, some equipment is particularly needed to enable the staff to visually see the status of the athletes. As a high-intensity exercise, basketball also requires wearable sensors to transmit the physical status of the athletes in real time [4]. The two-dimensional convolution kernel has more angles and can utilize more spatial dependence of sensor data [5]. Compared with the traditional HAR based on statistical analysis features and the HAR based on one-dimensional convolution kernel, the traditional two-dimensional convolution and method can improve the accuracy and accuracy of the evaluation index value, but cannot make full use of these three positions. It makes full use of the characteristics of the axis sensor data and the spatial dependence of the 3-axis accelerometer, so it will be applied to the sensor [6]. The diversification of sensor shapes can be divided into the diversification of sensor types and the diversification of sensor parts. The premise of the two-dimensional convolution and keying of the structure in the text is the diversification of sensor parts. The diversification of sensor types is based on the motion recognition of various types of sensors, such as the recognition of sensors such as acceleration sensors, mobile phone gyroscopes and magnetometers [7]. The diversification of sensor parts is based on the same type of sensor or the motion recognition of different types of sensors.

## 2. Related Work

Human behavior recognition uses one-dimensional sensor signals collected as a common multi-classification problem to extract discriminatory features, and finally uses machine learning methods to recognize human activities. The method of machine learning can usually be divided into supervised learning and unsupervised learning [8]. The so-called supervised learning means that we must label the data information that needs to be classified. The Literature combines the characteristics of data information and finds each category corresponding to the label [9, 10]. Therefore, for labeled data, the quality of the classification method plays an important role in the effectiveness of the human behavior recognition system. Traditional classification methods used to recognize human behavior include naive Bayes, svm algorithm, decision tree algorithm, nearest neighbor and hidden Markov entity model. LinSun et al. improved the SVM support vector machine to recognize 7 common human behaviors, and its accuracy increased from 91.5 to 93.1. Therefore, reference is very important to classify the traditional personal behavior of the supervised, and it closely combines the reasonable svm algorithm method with the excellent classification method [11]. Since the reference clearly proposed the definition of deep neural network, after more than ten years of development, deep neural network has become the most popular scientific research focus in the deep learning industry [12]. The essence of deep learning is that the role of each layer is not designed by human construction engineering, but a self-learning process based on data and information. Recent references have shown strong and excellent self-learning capabilities in many industries and directions, such as facial recognition, image recognition technology, speech recognition technology and natural language understanding solutions [13]. In the human behavior recognition industry, deep neural networks can be fully automated, collection improves the efficiency of human behavior recognition, and greatly simplifies complex engineering projects. Convolution and neural network is a deep feedforward control neural network algorithm, which has four core contents: local connection, weight value sharing resources, pool sampling and multi-solution layer accumulation, and used for image processing and Has excellent performance. Literature pointed out that convolutional neural networks are often used to study the recognition of human behavior [14]. At the same time, its research results also prove that convolutional neural networks are very suitable for processing human behavior recognition data [15]. Judging from the current development of human behavior recognition, the feature extraction step may no longer have major innovations and changes. Instead, choose to use convolutional neural networks to skip this step and let the computer automatically learn what it needs. The characteristics of behavior recognition have been the development requirements and trends. In our work, we use smart phone-based human daily behavior recognition as the background, and optimize the convolutional neural network to make it more suitable for behavior recognition data to carry out detailed experiments and comparative studies.

## 3. Research on CNN Multi-Position Wearable Sensors

### 3.1. One-Dimensional Convolution Kernel lD in HAR. cNN Application

**Figure 1:**
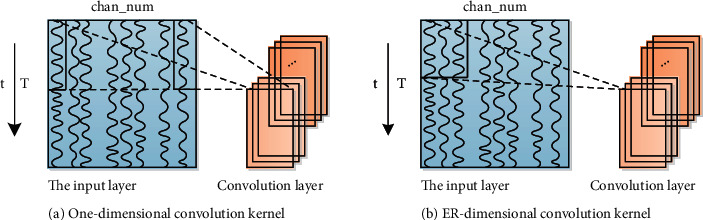
Convolution structure based on multi-channel time series data.

The one-dimensional convolution kernel is applied to one-dimensional time series data to extract time-related features. It is based on various sensors of smart wearable devices. However, sensor data information and sensor safety channel data information are all one-dimensional time series analysis data information. The time series data of each axis represents one channel.  Figure 1 describes the convolution method of most one-dimensional convolution kernels. The data includes sensor data with 3-axis attributes, and it may also include sensor data information that does not have 3-axis features, such as heartbeat. The keyin drainage matrix is a drainage matrix composed of the size of the sliding window *r* and the number of sensor safety channels. The one-dimensional convolution kernel method performs convolution operation on the data information of each safety channel, so it can automatically extract actions. When the sliding window is fixed, however, in some related literature, the selection of sensors is slightly different from the structure of CNN.

Generally, the input of the one-dimensional convolution kernel is usually one-dimensional initial data signal data information. The one-dimensional convolution kernel can better grasp the time dependence of the one-dimensional data signal in the entire process of the svm algorithm, but it often ignores different types of sensors or different axes of the same sensor that will damage people's cognition. The dependence of space on data information.

Compared with the one-dimensional convolution kernel that only considers the time domain dependency, the two-dimensional convolution kernel has more angles and can utilize more spatial dependency of the sensor data. Compared with the traditional HAR based on statistical analysis features and the HAR based on one-dimensional convolution kernel, the traditional two-dimensional convolution and method can improve the accuracy and accuracy of the evaluation index value, but cannot make full use of these three positions. It makes full use of the characteristics of the axis sensor data and the spatial dependence of the 3-axis accelerometer. Therefore, in this article, based on the data characteristics of the 3-axis sensor, we fully explore the temporal and spatial dependence of the same axis data and design a A new input organization form composed of multiple action graphs to enhance the recognition effect of HAR.

### 3.2. HAR Model of Multi-Position Three-Axis Sensor Based on Two-Dimensional Convolution Kernel

This article clearly proposes a HAR model for most three-axis smart wearable devices based on a two-dimensional convolution kernel. The physical model conforms to the two-dimensional convolution and definition in the HAR problem, but it improves the dependence of the time series analysis data information on the indoor space. Discovery. Therefore, in this article, everyone clearly proposes a new method of fusing the feature structure of the three-axis sensor into the posture image input by the two-dimensional convolution kernel, and a method to improve the data information of the same axis using the three-axis sensor. In the way of the axis sensor, different parts produce the typing posture interface. The original record is formed into 3 or 4 action pictures, and convolution and neuron networks are established according to the typed action pictures, and the time dependence of the data information on each axis of the original record is obtained, and there are many distinguishable matrix features. Value and identify the model, thereby improving accuracy. In order to simplify the model parameters and optimize the parameters of the convolutional network, we propose a HAR model based on shared parameters.

In other words, how to learn the main parameters of the entity model. The key issue is how to obtain high-resolution features from the initial label data. The key issue is how to construct action pictures for 2D convolution and core input. Due to the different organization of action pictures, the features acquired by 2D convolution and core will have different dependencies. Compared with one-dimensional time series data, moving pictures are two-dimensional signal data generated by performing dimensionality increase operations on one-dimensional signals through certain rules. The sliding window is the time window of the active photo and the time processing unit of the time series sensor data, and then:

(1) $$\begin{array}{c} M_{\left(A,B\right)}=argminLoss\left(A,B\left|D\right.\right). \end{array}$$

Among them, 4 is the main parameter drainage matrix of the weight value of the entity model *M* and the main parameter drainage matrix of the reference point. The culprit of the highest Dss is the loss function, that is to say, the function that causes the least loss function is the data information *D* entity The main parameters of the model are obtained under the standard.

The diversification of sensor shapes can be divided into the diversification of sensor types and the diversification of sensor parts. The premise of the two-dimensional convolution and keying of the structure in the text is the diversification of sensor parts. The diversification of sensor types is based on the motion recognition of various types of sensors, such as the recognition of sensors such as acceleration sensors, mobile phone gyroscopes and magnetometers. The diversification of sensor parts is based on the same type of sensor or the motion recognition of different types of sensors. This article synthesizes the characteristics of the three-axis diameter sensor, abandons the dependence of indoor space in the middle of the three axes of the same sensor, selects the dependence of the same shaft diameter data information of different sensors on the indoor space, and constructs the action of the convolutional network input Pictures, explain the basic principles of the construction of action pictures in the text from two aspects: basic theory and statistical analysis of data. The statistical analysis of the data is based on the basic theoretical analysis, and the data information of different shaft diameters of the same sensor and the original record of the same shaft diameter data information of the same sensor are analyzed. The square root of the correlation coefficient *r* below 0.3 is a weak correlation, and the weak correlation ratio of the correlation coefficient matrix is much higher than the ratio of the instantaneous velocity of each sensor on the same axis diameter of the sensor.

**Figure 2:**
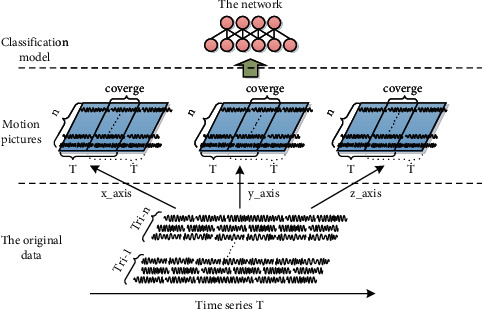
Multi-position three-axis sensor input construction method.

Figure 2 describes the motion image construction method based on the multi-position 3-axis sensor. The original time series data is obtained from the position 3-axis sensor, and 3 action pictures are used as the input of the 2D convolutional neural network. A position sensor that extracts the temporal and spatial correlations of different data on the same axis. Currently, the three-axis accelerometer is a relatively common sensor used in the process of human activity recognition. Most devices contain 3-axis accelerometers, such as bracelets, devices. Therefore, the three-axis sensor in this article uses a three-axis accelerometer.

Most devices have a built-in 3-axis accelerometer, but are also equipped with other sensors. Some of these sensors have 3-axis characteristics, such as 3-axis accelerometers, such as gyroscopes and magnetometers. Some are simple time series data, such as heart rate monitors. Therefore, considering this more practical and broader situation. Motion pictures use a one-dimensional convolution kernel instead of a two-dimensional convolution kernel in the convolution process, so only the time correlation of these time series data is extracted.

Algorithm 1 describes a method for constructing two-dimensional input moving images based on the multi-position hybrid sensor proposed in this paper. *M*. 2DCNN is an extension of T-2DCNN, except that the 4th and 7th rows of Algorithm 1 are added. The result returned by the algorithm is an action graph and corresponding labels after dividing the input data according to the size of the sliding window.

### 3.3. HAR Model Based on Two-Dimensional Convolution Kernel

**Figure 3:**
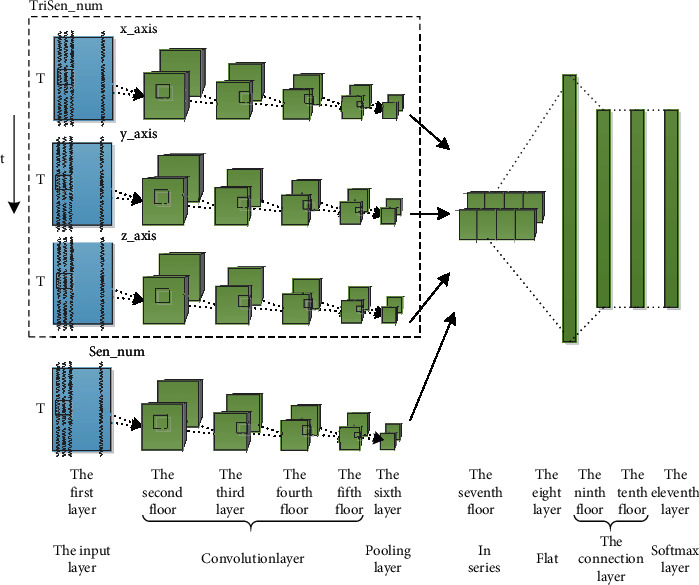
Two-dimensional convolution model network structure M.2DCNN based on multi-position hybrid sensor.

In the field of activity recognition based on sensor time series data, unlike traditional statistical functions, CNNs are widely used in this field because they can automatically extract high-resolution functions from raw data without requiring specialized domain knowledge. According to the composition of the action pictures, convolution and neural networks are used to obtain and classify features. The two-dimensional convolution and construction based on the multi-part hybrid sensor is an improved method based on the two-dimensional convolution and construction of the multi-part instantaneous velocity sensor. Therefore, Figure 3 represent the convolution and solid model of T-3DCNN and M.3DCNN Established, the 2D convolution operation of 2DCNN is in the dotted box.

### 3.4. Based on Parameter Sharing Two-Dimensional Convolutional Network Structure

**Figure 4:**
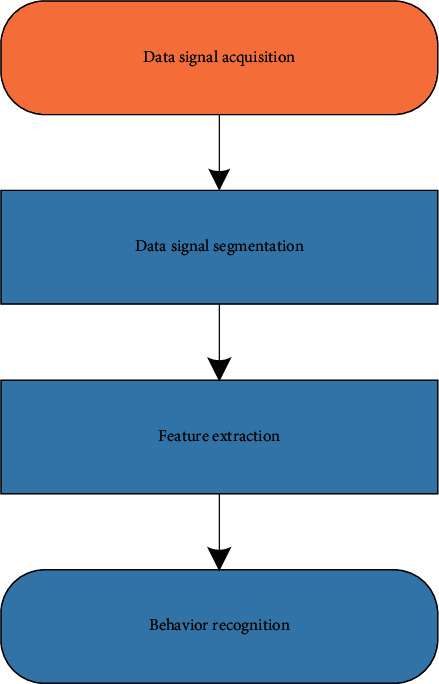
Two-dimensional convolution calculation process.

Compared with the traditional fully connected network, the convolutional network significantly reduces the number of parameters, but during the training process of M.2DcNN and T.2DcNN, each action picture requires a separate convolution operation, so the convolution operation will still generate a lot of parameter. Two-dimensional convolution calculation process as show in Figure 4.

Therefore, in the convolution process of each moving image, the parameters that need to be trained between the Z-1th layer and the first layer are given by formula (4).

(2) $$\begin{array}{c} P_{num}=K_{n}^{l-1}\times K_{n}^{l}\times K_{h}^{l}\times K_{w}^{l}+K_{n}^{l}. \end{array}$$

According to the convolutional network training model shown in the figure above, the model parameters are independent in the convolution operation of each action picture, and the number of parameters increases as the size of the convolution kernel and the number of feature maps increase. Therefore, this paper proposes a convolutional network model TS based on shared convolutional layer parameters. The parameters are based on T-2DcNN and M.2DCNN. By combining the attributes of the shorthand data of the action pictures and the attributes of the model, we will Use the same parameter set to perform feature extraction on three sets of action pictures. The ability to share parameters is that three different sets of parameters are trained in the convolution operation. The same parameter set can be used for the purpose of extracting data features from various directions, thereby reducing the number of training parameters and ensuring the recognition effect. Comparison of the number of convolutional layer parameters is as shown in Table 1.

## 4. Research Based on Basketball Training

### 4.1. Model Training and Implementation

This article uses two-dimensional convolution kernel and one-dimensional convolution sum, and the calculation of the two-dimensional convolution sum in the whole process of forward expansion is connected, as shown in formula (3).

(3) $$\begin{array}{c} z_{i,j}^{k}=\overset{K_{n}^{l-1}}{\underset{k^{l-1}=1}{\sum }}\overset{K_{h}^{l}}{\underset{u=1}{\sum }}\overset{K_{w}^{l}}{\underset{v=1}{\sum }}a_{u,v}^{\left(k^{l-1},k^{l}\right)}x_{\left(i-1\right)\cdot h_{s}^{l}+u,\left(j-1\right)\cdot w_{s}^{j}+v}^{k^{l-1}}+b_{k^{l}}. \end{array}$$

Among them, *z* represents the mapping coordinate input value of the z-th layer.

(4) $$\begin{array}{c} k^{l}=\left[1,K_{n}^{l}\right],a_{u,v}^{\left(k^{l-1},k^{l}\right)}. \end{array}$$

The first function map value of the first layer:

(5) $$\begin{array}{c} x_{\left(i-1\right)\cdot h_{s}^{l}+u,\left(j-1\right)\cdot w_{s}^{j}+v}^{k^{l-1}}. \end{array}$$

The first feature mapping coordinates are:

(6) $$\begin{array}{c} \left(\left(i-1\right)\cdot h_{s}^{l}+u,\left(j-1\right)\cdot w_{s}^{j}+v\right). \end{array}$$

The output value of the second function chart:

(7) $$\begin{array}{c} x_{i,j}^{k^{l}}=\sigma \left(z_{i,j}^{k^{l}}\right). \end{array}$$

In the pooling layer (that is, the sub-sampling layer), the maximum value of all nerve cells in the sub-region of the convolutional layer is selected to generate the pooling layer nerve cells. In the neural network structure of this article, there are two cases, the convolutional layer connected to the convolutional layer and the convolutional layer connected to the pooling layer. If the *Z* layer is a convolutional layer, the residual term of the first mapping layer is:

(8) $$\begin{array}{c} \delta_{i,j}^{k^{l-1}}=\frac{\partial L}{\partial z^{k^{l-1}}}=\overset{K_{n}^{l}}{\underset{k^{l}=1}{\sum }}\frac{\partial L}{\partial z^{k^{l-1}}}\cdot \frac{\partial z^{k^{l}}}{\partial z^{k^{l-1}}}=\overset{K_{n}^{l}}{\underset{k^{l}=1}{\sum }}\delta_{i,j}^{k^{l}}\cdot \frac{\partial z^{k^{l}}}{\partial z^{k^{l-1}}}\\ =\overset{K_{n}^{l}}{\underset{k^{l}=1}{\sum }}\delta_{i,j}^{k^{l}}⊗rot180\left(a^{k^{l-1},k^{l}}\right)\cdot \sigma^{\prime }\left(z^{k^{l-1}}\right). \end{array}$$

After obtaining the formula for calculating the residual term, the formula for the slope is:

(9) $$\begin{array}{c} \frac{\partial L}{\partial a_{u,v}^{\left(k^{l-1},k^{l}\right)}}=\overset{h^{l}}{\underset{i=1}{\sum }}\overset{w^{l}}{\underset{j=1}{\sum }}x_{\left(i-1\right)\cdot h_{s}^{l}+u,\left(j-1\right)\cdot w_{s}^{j}+v}^{k^{l-1}}\cdot \delta_{i,j}^{k^{l}}. \end{array}$$

Entity model completion optimization algorithm 2 describes the whole process of HAR model training based on two-dimensional convolution and moving images. For each action picture, 4 convolutions and 1 unified actual operation are performed, and feature projection is performed. Solving patchwork and floor plans, the seventh and eighth lines match the actual operation of the fully connected layer and the output layer, and the input of the fully connected layer is the eigenvalues of the one-dimensional matrix solved on the sixth network, and the 9th line includes all layers Back propagation measurement. If the main parameter conversion is less than the threshold or all data information is trained, the main parameters of the physical model can be obtained. Pay special attention to the whole process of specific calculation, divide the training data information into several rounds, and calculate in the form of drainage matrix.

### 4.2. Experiment and Analysis

This article compares the gesture recognition effects of two conventional feature extraction algorithms on public data sets, such as the traditional 2D convolution input 1DCNN and the traditional 2D convolution input 2DCNN. In addition, the reason for choosing lDCNN and 2DCNN is that the focus of this article is not to detect the current CNN network architecture and the hybrid entity model that mixes CNN and different classification models, but only cares about the verification of the actual effect of the svm algorithm. At the same time, all classification models use softIIl for classification.In the field of human activity recognition based on sensor signals, public data sets used for testing are usually divided into two categories based on the location of sensors. Sensor data (for example, by placing sensors on items such as drawers and cups, and combining sensors on Physically to recognize movement), the public data set used in this article belongs to the first type of data oppornmty data set and SKODA data set.

In this article, we use body sensors to identify the movement of the tester, including 7Imu and 12 3-axis acceleration sensors. The distribution is shown in Table 2. For each tester, six documents will be recorded, including five ADLs and one exercise. ADL recorded the theme activities of the test engineer in the natural environment, including lying on the table and chair, running around the room, preparing freshly ground coffee, sandwiches and other activities in advance, including opening and closing the refrigerator and opening the refrigerator., Such as closing the drawer. In the case of the activity category, the data is divided into multiple levels, divided into gesture pattern recognition and gesture recognition, and gesture recognition is divided into high-level activities, intermediate activities and sub-activities. Higher-level activities are abstract activities, such as coffee breaks, Sandwich breaks. Intermediate activities are a complete high-level activity. For example, coffee breaks include activities such as opening and closing drawers, and low-level activities are the unit of activity for intermediate activities. In this article, middle-level exercise is selected as the identification tag, and the motion pattern recognition describes the motion state, such as standing, lying down, and sitting. Opportunity and SKODA data set is as shown in Table 3.

The experimental natural environment in this article is the GPU produced by Googlecolaborato, the computer language is *Python*2, and the architecture is Kefas.  Table 4 lists the main parameters required during the test. In order to better verify the characteristics of the optimization algorithm under various sliding windows, the main parameter categories of the sliding window are designed. The main parameter of the dialog box is 24, that is, 24 time data information is used as the data unit for training and predictive analysis, and the final time data information of the dialog box is used as the identification of the data unit. The popularity rate of the dialog box is 50%. Because the SKoDA sampling rate is very high, the SKODA data information will be down-sampled to complete the shared resources of the main parameters of the test and facilitate comparison with OPPORTuNITY data. When the sliding window is 24, Table 4 shows the number of training samples of OPPORTUNITY and SKODA data, and identifies various symbols.

In addition, from the perspective of the total number of sensors, the actual effects of the svm algorithm of the convolutional network entity model and the main parameter entity model of shared resources are verified. For OPPORTI elbow data, some IMU sensors are reasonable. There are 4 groups of sensors in the whole body, and the default setting is that the last group includes 16 kinds of sensors. Similarly, for sKoDA data, if it is added according to the area where the sensor is located, it is divided into 3 groups. The first group contains 3 types of forearm sensors, the second group contains 8 types of large arms and forearms, and the last group contains 10 types. In the references using OPPORTIITY and SKODA data, due to basic parameters and precise measurement specifications Different, so it is impossible to make a unified accurate measurement. Therefore, the reference method is integrated into the text, and the main parameters of the input data information for more experiments are integrated.

(10) $$\begin{array}{c} F_{I}=\overset{C}{\underset{i=1}{\sum }}F_{i}=\overset{C}{\underset{i=1}{\sum }}w_{i}\cdot \frac{2P_{i}+R_{i}}{P_{i}+R_{i}}. \end{array}$$

### 4.3. Sensor Design

Data acquisition, also known as data acquisition, refers to the process of collecting signals from sensors and other devices under test. Data collection technology is widely used in various fields, and human behavior recognition is one of them. There are different types of collection tools for collecting data, such as different sensor devices, such as cameras and microphones. At present, smart devices integrate many embedded sensors for collecting data and information, which provide great convenience for the research of human behavior recognition based on smart devices, and they are also included in the field of human behavior recognition. The very broad application prospects and the role of built-in sensors in large-scale advertising in smart devices are particularly important in this topic.

An acceleration sensor is an electronic device that can measure the acceleration force generated by an object when it moves. It is the force acting on the object itself when the object is accelerating. If the smart device is fixed on the left side of the horizontal plane, the acceleration on the *y* and *z* axes are both zero, and the *x*-axis will be statically affected by the gravity of the smart device. At this time, since the *x*-axis is positive, the gravitational acceleration of the smart device is negative, and the *x*-axis acceleration is as follows.

(11) $$\begin{array}{c} a_{x}=a-\left(-g\right)=a+g. \end{array}$$

Therefore, when the left side of the smart device is placed on a horizontal surface, the acceleration on the *x*, y, and *z* axes is similar to:

(12) $$\begin{array}{c} \left(a_{x},a_{y},a_{z}\right)=\left(g,0,0\right). \end{array}$$

Conversely, if the device is placed in a horizontal plane with the right side of the device facing down and the *x*-axis direction down, the acceleration due to gravity is +*g*. In this case, the acceleration on the *x*-axis is:

(13) $$\begin{array}{c} a_{x}=a-g. \end{array}$$

Approximate values at rest:

(14) $$\begin{array}{c} \left(a_{x},a_{y},a_{z}\right)=\left(-g,0,0\right). \end{array}$$

If our device is facing up and placed perpendicular to the horizontal plane, the acceleration on the *x* and *z* axis is zero, while the *y* axis is fixed by its own gravity. Since the upward direction of the *y*-axis is positive and the downward direction is negative, the gravitational acceleration of the smart device is negative, and the acceleration on the *y*-axis is:

(15) $$\begin{array}{c} a_{y}=a-\left(-g\right)=a+g,\\ \left(a_{x},a_{y},a_{z}\right)=\left(0.g,0\right). \end{array}$$

On the contrary, if we place the device with the head of the smart device facing down and the *y*-axis direction down, the acceleration of gravity is +*g*. In this case, the acceleration of the *y*-axis:

(16) $$\begin{array}{c} a_{y}=a-g. \end{array}$$

Approximate values at rest:

(17) $$\begin{array}{c} \left(a_{x},a_{y},a_{z}\right)=\left(0,-g,0\right). \end{array}$$

If the screen of the smart device is placed on a horizontal surface, the *x*-axis and *y*-axis acceleration are 0, and the positive direction of the *z*-axis is upward, and the gravitational acceleration is negative, so the *z*-axis acceleration is:

(18) $$\begin{array}{c} a_{z}=a-\left(-g\right)=a+g. \end{array}$$

Therefore, when the device's screen is placed on a horizontal surface, the 3-axis acceleration of the smart device is similar to:

(19) $$\begin{array}{c} \left(a_{x},a_{y},a_{z}\right)=\left(0,0,g\right). \end{array}$$

Conversely, if the device screen is flat relative to the horizontal plane and the positive *z*-axis is facing down, the acceleration due to gravity is +*g*. In this case, the acceleration on the *z* axis is:

(20) $$\begin{array}{c} a_{z}=a-g. \end{array}$$

Approximate values at rest:

(21) $$\begin{array}{c} \left(a_{x},a_{y},a_{z}\right)=\left(0,0,-g\right). \end{array}$$

### 4.4. Factors Affecting the Quality of Basketball Training

Basketball is very antagonistic. It requires athletes to have strong physical fitness, strength and stamina to ensure that their technical movements will not be deformed. Therefore, having good physical stamina is an important basis for basketball. In physical education, many students are not professional athletes, and they also lack stable exercise. Therefore, in physical training of basketball in physical education, many students will have problems with physical training due to poor physical fitness.

Many students who choose to participate in basketball have no real goals. Some students choose to participate in the sport blindly and are not interested in the sport. These students not only do not understand sports, but also lack basketball knowledge. When doing basketball exercises for these students, they are easily frustrated due to limited endurance, and physical training is relatively cumbersome and easily produces resistance. If the mental state is not well controlled, then basketball fitness training is difficult to proceed smoothly. Therefore, it is very important to adjust the mental state of students in the actual training process. People can use small games or encouraging words to enhance students' confidence, stimulate students' enthusiasm for participation, and improve efficiency.In basketball physical exercise, students should be divided into several categories according to their actual physical conditions, and the training methods for each group should be formulated reasonably so that students will not encounter situations where their physical strength cannot adapt to the training content. If the teaching content is beyond the acceptable range of the students, not only will the effect be poor, but it will also bring pressure to the students and even make them feel resistant. Due to the relatively high intensity of physical training, it is very easy to cause student fatigue. To avoid this situation, you must appropriately extend the training interval, but this will not achieve the desired effect of training, so it is scientific and reasonable Local grouping and arrangement of training content are very important.

In basketball physical exercises, special psychological adjustments should be made appropriately to enhance students' self-confidence and patience, and stimulate their enthusiasm for learning. In particular, swimming can be added to physical exercise, and swimming training can improve students' cardiorespiratory function and increase endurance, and help students learn how to overcome fear and enhance self-confidence. Some students who perform well in physical education courses should be appropriately praised, especially in endurance training, teachers should give appropriate encouragement to keep students patient, students with poor physical fitness should have corresponding encouragement and objective opinions. Effectively stimulate students' enthusiasm for participation through student achievements so that others can recognize their efforts.

## 5. Conclusion

In this article, the traditional two-dimensional convolution and keying method cannot explore the dependence of the coaxial data information of the multi-part three-axis sensor on the indoor space, so scientific research and solutions are clearly proposed. The document clearly proposes two network architectures, namely T-3DCNN and M-3DCNN. The key is to apply the T.3D and M.3D construction methods to divide the data information of the three-axis acceleration sensor from several parts into three separate areas in order to enter the two-dimensional convolution kernel and form a posture of the non-three-axis sensor. One-dimensional convolution and core type images, according to the composition of feature maps obtained from multiple pose images, obtain high-dimensional spatial features, and the method clearly proposed by this application is completed as the HAR model of the svm algorithm method. In addition, according to the network architecture of the main parameters Ts.cNN and Ms.3DcNN of shared resources, the network architecture ensures the accuracy of recognition. In addition, it reduces the total number of training main parameters in the convolutional layer, and sets various test main parameters in the authentication text to the actual effect of the above method. In future research work, the actual environment modeling and behavior perception will be combined with the data collected by the project research team.

## Figures and Tables

**Algorithm 1:**
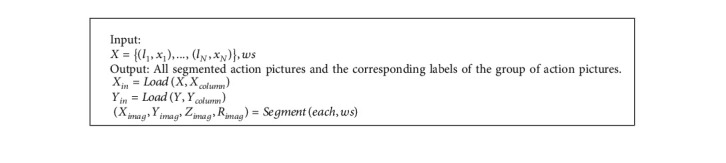
Two dimensional input algorithm of moving image based on multi position hybrid sensor.

**Table 1:** Comparison of the number of convolutional layer parameters.

Convolutional layer	Up one level	Method	
		T-2DCNN/M-2DCNN	TS-2DCNN/MS-2DCNN
Cov2_X	Input_X	3 × 3 × 64 + 64 = 640	640
Cov2_Y	Input_Y	640	
Cov2_Z	Input Z	640	

Cov3_X	Cov2_X	3 × 3 × 64×64 + 64 = 36928	36928
Cov3_Y	Cov2 Y	36928	
Cov3_Z	Cov2 Z	36928	

Cov4_X	Cov3_X	5 × 1 × 64×64 + 64 = 20544	20544
Cov4_Y	Cov3_Y	20544	
Cov4_Z	Cov3_Z	20544	

Cov5_X	Cov4_X	20544	20544
Cov5_Y	Cov4_Y	20544	
Cov5_Z	Cov4_Z	20544	

Total		235968	78656

**Table 2:** Opportunity data set sensor location distribution.

Position	IMU	Accelerometer (ACC)
Left arm	LUA, LLA	Lua, LUA_, LWR, LH
Right arm	RUA, RLA	RUA_, RUAA, RWR
Left leg	LSHOE	
Right leg	RSHOE	RKNA, RKN_
Main trunk	BACK-I	BACK-a, HIP

**Table 3:** Opportunity and SKODA data set description.

OPPORTUNITY						SKODA		
GR			Lm					
Label	Quantity	Symbol	Label	Quantity	Symbol	Label	Quantity	Symbol
Close dishwasher	716	S1	Stand	22388	L1	Write on notepad	1386	S1
Close drawer 3	624	S2	Walk	13183	L2	Open hood	1643	S2
Close drawer 2	444	S3	Sit	9427	L3	Close hood	1540	S3
Close door 1	871	S4	Lie	1674	L4	Check gaps (front door)	1141	S4
Close door 2	927	S5	Null	693	L0	Open left front door	675	S5
Close drawer 1	453	S6				Close left front door	639	S6
Close fridge	1010	S7				Close both left door	1188	S7
Toggle switch	740	S8				Check trunk gaps	1315	S8
Open dishwasher	765	S9				Open and close trunk	1573	S9
Open drawer 3	634	S10				Check steering wheel	884	S10
Open drawer 2	506	S11						

**Table 4:** Experimental parameters.

	Parameter	Default value
Sliding window	24, 48	Twenty-four

OPPORTUNITY	Category 8: ACC (right arm) and IMU (left arm, right arm, main trunk)	Class 16
	10 categories: ACC (right arm, right yao) and IMU (left arm, right arm, main trunk)	
	14 categories: ACC (right arm, right leg, left bone) and IMU (left arm, right arm, main trunk)	
	Class 16: ACC (right arm, right leg, left all, main trunk) and IMU (left arm, right arm, main trunk)	

Sensor category	Category 3 : 2, 27, 16 (forearm)	10 categories
	8 categories 227, 16, 18, 14, 242221 (small arm, big arm)	
	10 categories: 2, 27, 16, 18, 1424, 22, 21, 29, 1 (forearm, big arm, elbow)	

SKODA	Layer 2 : 3×3 and 3×1 layer 3 : 3×3 and 3×*l*	Same parameter
	Layer 4 : 5×*l*	
	Floor 5 : 5×*l*	
	Layer 6 : 3×*l*	

Sensor category	0.01	Same parameter

Convolution kernel	1	Same parameter

## Data Availability

The data used to support the ﬁndings of this study are available from the corresponding author upon request.
